# Is Low Non-Lethal Concentration of Methylmercury Really Safe? A Report on Genotoxicity with Delayed Cell Proliferation

**DOI:** 10.1371/journal.pone.0162822

**Published:** 2016-09-13

**Authors:** María Elena Crespo-Lopez, Allan Costa-Malaquias, Edivaldo H. C. Oliveira, Moysés S. Miranda, Gabriela P. F. Arrifano, José R. Souza-Monteiro, Fernanda Espirito-Santo Sagica, Enéas A. Fontes-Junior, Cristiane S. F. Maia, Barbarella M. Macchi, José Luiz M. do Nascimento

**Affiliations:** 1 Laboratório de Farmacologia Molecular, Instituto de Ciências Biológicas, Universidade Federal do Pará, 66075-110 Belém (Pará), Brasil; 2 Laboratório de Cultura de Tecidos e Citogenética, Departamento de Meio Ambiente, Instituto Evandro Chagas, 67030-000 Ananindeua (Pará), Brasil; 3 Laboratório de Fertilização In Vitro, Instituto de Ciências Biológicas, Universidade Federal do Pará, 66075-110 Belém (Pará), Brasil; 4 Laboratório de Farmacologia da Inflamação e do Comportamento, Instituto de Ciências da Saúde, Universidade Federal do Pará, 66075-110 Belém (Pará), Brasil; 5 Laboratório de Neuroquímica Molecular e Celular, Instituto de Ciências Biológicas, Universidade Federal do Pará, 66075-110 Belém (Pará), Brasil; Chinese Academy of Sciences, CHINA

## Abstract

Human exposure to relatively low levels of methylmercury is worrying, especially in terms of its genotoxicity. It is currently unknown as to whether exposure to low levels of mercury (below established limits) is safe. Genotoxicity was already shown in lymphocytes, but studies with cells of the CNS (as the main target organ) are scarce. Moreover, disturbances in the cell cycle and cellular proliferation have previously been observed in neuronal cells, but no data are presently available for glial cells. Interestingly, cells of glial origin accumulate higher concentrations of methylmercury than those of neuronal origin. Thus, the aim of this work was to analyze the possible genotoxicity and alterations in the cell cycle and cell proliferation of a glioma cell line (C6) exposed to a low, non-lethal and non-apoptotic methylmercury concentration. Biochemical (mitochondrial activity) and morphological (integrity of the membrane) assessments confirmed the absence of cell death after exposure to 3 μM methylmercury for 24 hours. Even without promoting cell death, this treatment significantly increased genotoxicity markers (DNA fragmentation, micronuclei, nucleoplasmic bridges and nuclear buds). Changes in the cell cycle profile (increased mitotic index and cell populations in the S and G2/M phases) were observed, suggesting arrest of the cycle. This delay in the cycle was followed, 24 hours after methylmercury withdrawal, by a decrease number of viable cells, reduced cellular confluence and increased doubling time of the culture. Our work demonstrates that exposure to a low sublethal concentration of MeHg considered relatively safe according to current limits promotes genotoxicity and disturbances in the proliferation of cells of glial origin with sustained consequences after methylmercury withdrawal. This fact becomes especially important, since this cellular type accumulates more methylmercury than neurons and displays a vital role protecting the CNS, especially in chronic intoxication with this heavy metal.

## Introduction

Mercury exposure is a serious public health problem worldwide. In 2013, Brazil, along with 91 countries, signed the Convention of Minamata (www.mercuryconvention.org), with the aim of reducing and combating environmental and human exposure to this metal. This action was already recognized and supported by the World Health Organization with a resolution adopting the Convention [1]. Present safety limits established for human exposure by international agencies were mainly based on acute outbreaks, as in Minamata and Iraq [2–4]. However, in recent decades, concerns have been raised, since chronic exposure to relatively low levels of methylmercury (MeHg), the most toxic compound of mercury, can be found in regions as the Seychelles or the Amazon Basin, with contaminated fish as the main source responsible for human exposure [5–7]. This type of intoxication activates several cellular mechanisms that can potentially lead to long-term deleterious consequences, most importantly genotoxicity [8, 9]. It is currently unknown as to whether exposure to low levels of mercury below established limits is safe.

Low concentrations of mercury have already been demonstrated to have deleterious effects by provoking significant genotoxicity in primary cultures of human lymphocytes [8]. However, studies with cells of CNS, which the main target of MeHg, are sparse. Interestingly, cells of glial origin are able to accumulate higher concentrations of MeHg when compared to cells of neuronal origin [10], showing more intense damage to DNA such as nucleoplasmic bridges and an increased number of micronuclei per cell [11]. These effects could be accompanied by alterations in the cell cycle and/or the ability of the cell to adequately proliferate. Disturbances to the cell cycle and cellular proliferation due to MeHg exposure have already been observed for cells of neuronal origin [12–15]. However, no data are presently available about the effect of low levels of methylmercury on glial cells.

Thus, the aim of this work was to investigate the exposure of cells of glial origin to a low, non-lethal, non-apoptotic MeHg concentration and to analyze possible genotoxicity in the absence of cellular death and the possible alterations of cell cycle and cell proliferation accompanying this genotoxicity.

## Materials and Methods

### Cells and Treatments

**Fig 1:**
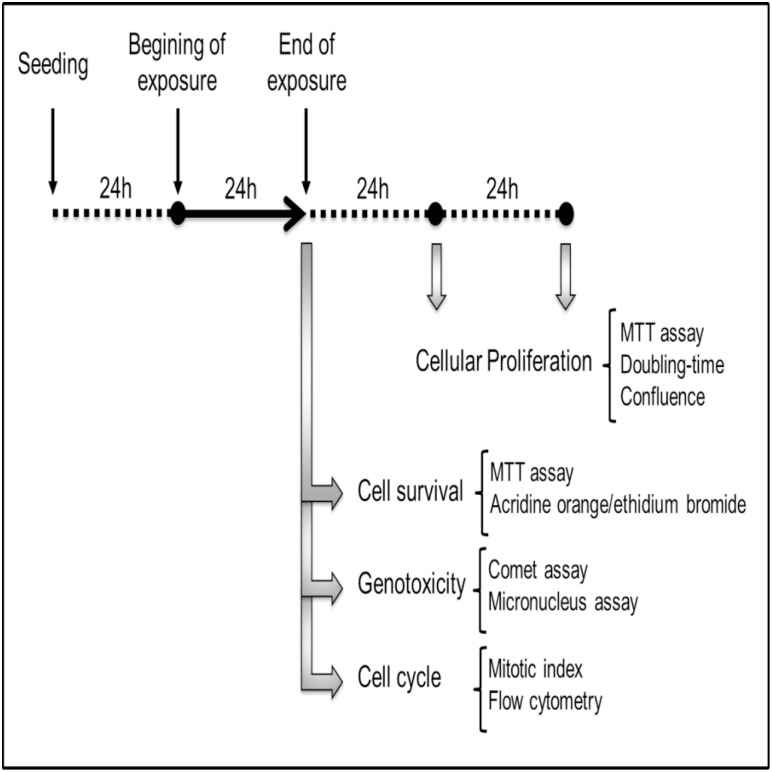
Time-line of the experimental design of this study including analysis carried out at each point.

The rat glioma C6 cell line (American Type Culture Collection, Manassas, VA) was maintained at 37°C and 5% CO_2_ in DMEM with 10% fetal bovine serum (FBS), penicillin (50 U/ml) and streptomycin (50 μg/ml). Approximately 1.5x10^5^ cells were seeded and maintained at 37°C for 24 h before exposure to methylmercury (MeHg). Cells were incubated with MeHg at a final concentration of 0–10 μM in DMEM supplemented with 10% FBS. After MeHg exposure, the culture medium was replaced with fresh medium without MeHg for 24 or 48 hours, followed by cell proliferation analysis, according to the experimental design (Fig 1).

### Cellular Viability Assay

Cellular viability was quantified via the 4,5 dimethylthiazol-3,5-diphenyltetrazolium (MTT) method [16]. After treatments, cells were washed twice with phosphate-buffered saline (PBS) and incubated for 2 h with MTT (0.5 mg/ml). The absorbance of the samples was recorded at 570 nm and cell viability was reported as the percentage of reduced MTT compared to that of the control group.

### Analysis of Apoptosis and Necrosis

After MeHg exposure, apoptosis/necrosis detection was carried out by acridine orange:ethidium bromide (AO:EB) double staining for fluorescent microscopy [17]. Cells were suspended in DMEM and gently mixed with the AO:EB staining solution (1:1). At least, four independent experiments were carried out and 300 cells per group were randomly selected and analyzed by fluorescence microscopy. The results were expressed as the percentage of viable or non-viable cells.

### Comet Assay

Comet assay (or single cell gel electrophoresis) was used to detect DNA lesions as strand breaks [18]. After treatment, cells were washed with Hank’s buffer and embedded in 0.5% low melting point agarose. Then, they were transferred onto agarose-precoated slides and incubated in a lysis solution containing 1% Triton X-100, 10% DMSO, 2.5 M NaCl, 100 mM EDTA, 10 mM Tris-HCl, pH 10. DNA unwinding was carried out in electrophoresis buffer (300 mM NaOH, 1 mM EDTA, pH 13) for 20 minutes at 4°C. Then, electrophoresis was performed (1 V/cm^2^, 300 mA) for 20 minutes and slides were neutralized with 19.5 mM Tris-HCl (pH 7.5). Slides were fixed, air-dried and stained with DAPI (10 μg/ml). Six independent experiments were carried out and a minimum of 100 cells per group (randomly selected) were analyzed using Comet 6.0 software (Andor, UK) and the results were expressed as the percentage of DNA in the head (non-fragmented) or in the tail (fragmented).

### Cytokinesis-block Micronucleus Assay (CBMN)

CBMN was carried out as described elsewhere [11] with adaptations. Briefly, after exposure, cells were incubated with cytochalasin B (8 μg/ml) for 30 h, fixed with methanol:acetic acid (3:1) solution and stained with 3% Giemsa. Six independent experiments (n = 6) were carried out and a minimum of 1000 cells/group (randomly selected) in each experiment were analyzed. Total number of cells (N), binucleated cells (B) and cells with micronucleus/micronuclei (M), nucleoplasmic bridges (NBr) or nuclear buds (NBu) were scored. Results were expressed as the binucleation index (B/N), micronucleus index (M/N), nucleoplasmic bridge index (NBr/N) and nuclear bud index (NBu/N).

### Analysis of the Mitotic Index

This analysis was performed as described before [9] with adaptations. After MeHg treatment, cells were incubated with colchicine (16 μg/ml) for 4 h, fixed with methanol:acetic acid (3:1) solution and stained with 3% Giemsa. The results were expressed as the number of metaphases in 1000 cells.

### Cell Cycle Analysis

Analysis of the cell cycle was performed by flow cytometry (FACS Canto II, Becton Dickinson, San José, CA, USA) following cell DNA staining with propidium iodide and using FACSDiva software (Becton Dickinson, San José, CA, USA). After intoxication with 3 μM of MeHg for 24 h, 3 x 10^6^ cells were pelleted and fixed in 1% ice-cold formaldehyde for 20 min at 4°C. Ice-cold methanol was added and the cells were placed at −20°C for 10 min. The cells were washed twice in PBS and resuspended in 20 μg/ml of propidium iodide for 30 min at 37°C in the dark and then they were analyzed by flow cytometry.

### Cellular Proliferation Analysis

Cellular proliferation, 24 h and 48 h after the end of the exposure, was studied by assessing cellular viability (analyzed by MTT assay as described above), determining the number of viable cells using trypan blue dye exclusion [19], by calculating cell doubling time [20] and by performing a visual evaluation of confluence by microscopy. MTT assay is a recognized method for cellular viability based on mitochondrial activity. Trypan blue method was used to know the exact number of cells for calculating the cell doubling time and to confirm MTT results. The results were expressed as the percentage of reduced MTT compared to that of the control group, the number of viable cells, the doubling time and by representative micrographs of cultured cells.

### Statistical Analysis

Student’s t-test and one-way ANOVA followed by the Tukey *post hoc* test when appropriate were used to compare two and more than two groups, respectively. The Pearson correlation was applied to calculate LC_50_. P<0.05 was considered statistically significant.

## Results

**Fig 2:**
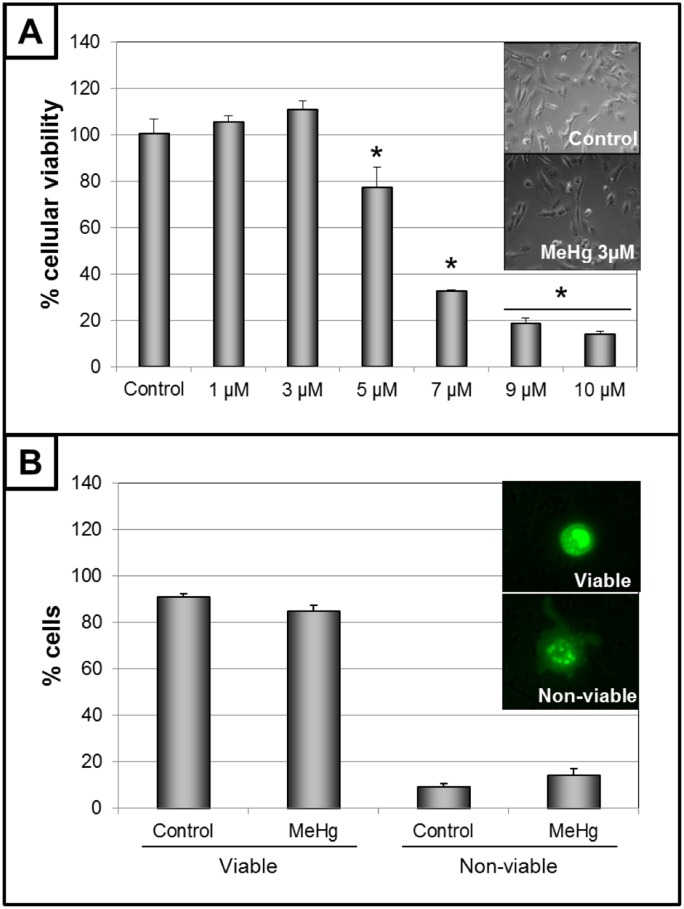
Cell survival after exposure to methylmercury (MeHg) for 24h. Cellular viability of cells intoxicated with increased concentrations (panel A) and number of viable and non-viable cells in control and cells exposed to 3 μM (panel B). Insets show micrographs (40X and 100X). Data are expressed as mean ± SEM (n = 4–9). One-way ANOVA followed by *post-hoc* Tukey test (panel A) and Student’s t-test between control and MeHg groups (panel B) were performed. *P < 0.01 *vs* all groups.

**Fig 3:**
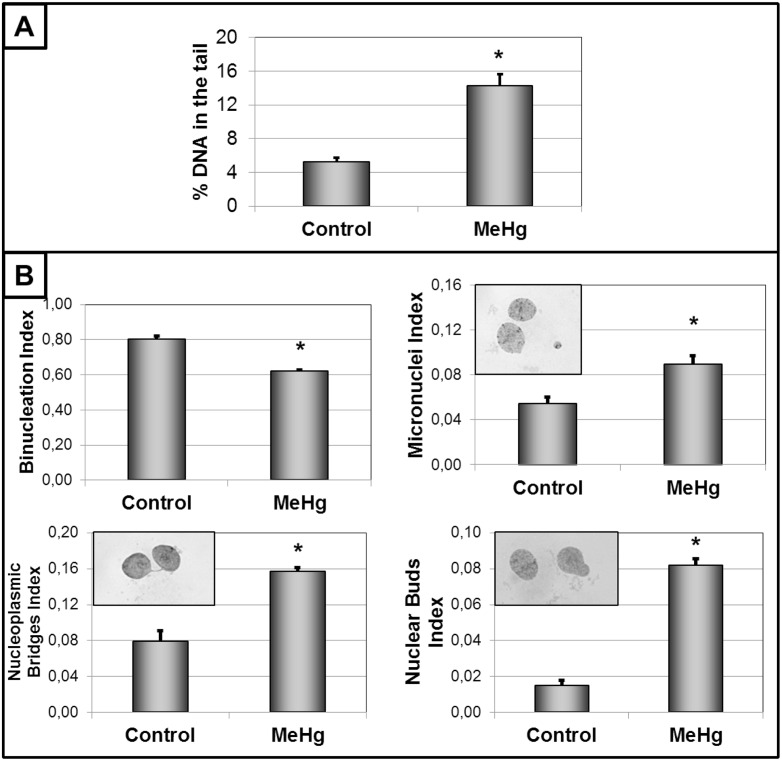
Genotoxicity detected after 24h of exposure to 3 μM of methylmercury (MeHg). DNA fragmentation was analyzed by comet assay (panel A) and indexes of micronuclei, nucleoplasmic bridges and nuclear buds were analyzed by cytokinesis-block micronucleus assay (panel B). Insets show micrographs (100X). Data are reported as mean ± SEM (n = 6). Student’s t-test between control and MeHg groups was performed. *P < 0.01 *vs* control.

**Fig 4:**
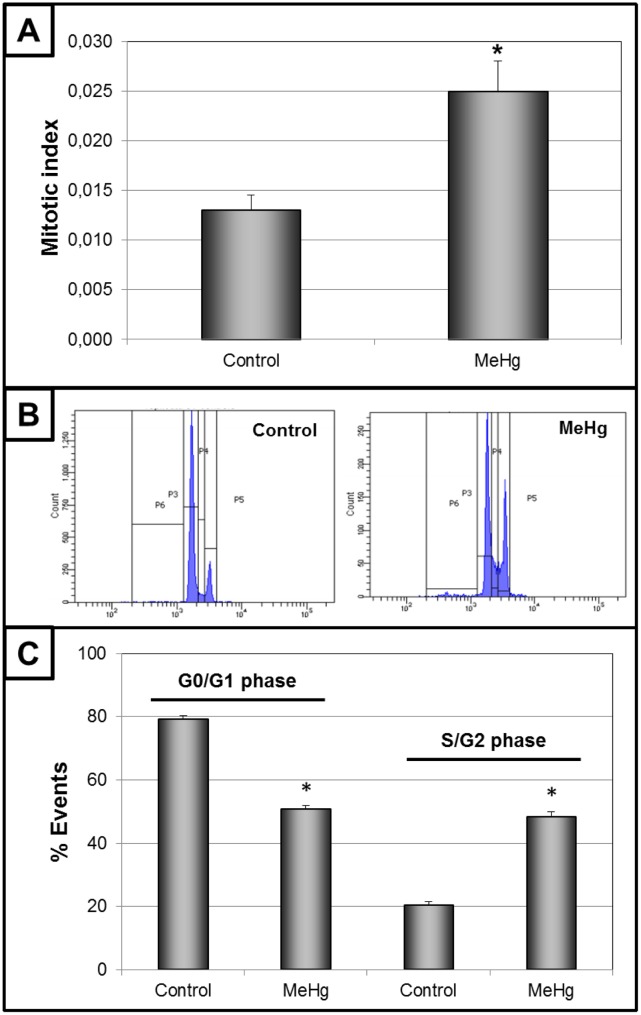
Alterations of the cell cycle after intoxication with 3 μM of methylmercury (MeHg) for 24h. Mitotic index (panel A) and cell cycle profile with illustrative spectrograms (panel B) and proportion of events (panel C). Data are shown as mean ± SEM (n = 3). Student’s t-test between control and MeHg groups was performed. *P < 0.01 *vs* control.

**Fig 5:**
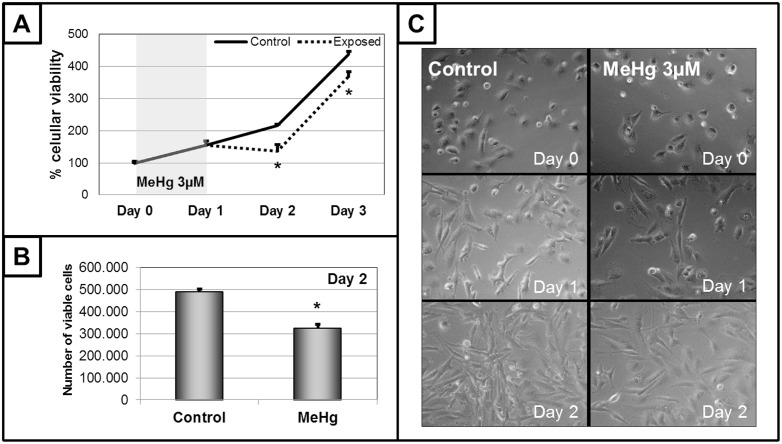
Cell proliferation after exposure to methylmercury (MeHg) 3 μM for 24h. Cellular viability was evaluated at the beginning (day 0) and the end (day 1) of exposure and after 24h (day 2) and 48h (day 3) of MeHg withdrawal (panel A). Number of viable cells were registered on day 2 (panel B). Micrographs (40X) of culture confluence at days 0, 1 and 2 are shown (panel C). Data are shown as mean ± SEM (n = 3–9). Student’s t-test between control and MeHg groups of the same day was performed. *P < 0.01 *vs* control group of the same day.

Cells exposed to increasing concentrations of MeHg for 24 h showed a concentration-dependent loss of cellular viability (Fig 2, upper panel) with an LC_50_ of 6.08 μM. Based on these results, 3 μM MeHg was the selected concentration used for the subsequent experimental characterization of MeHg-induced genotoxicity in the absence of cell death in cells of glial origin. Also, no significant apoptosis or necrosis was caused by exposure to this concentration of MeHg for 24 h resulting in similar numbers of viable and non-viable cells (Fig 2, lower panel). However, significant DNA fragmentation was detected by the comet assay, with less DNA detected in the head and more in the tail, after 24 h of intoxication with 3 μM MeHg (Fig 3, upper panel). This deleterious effect on DNA was confirmed by CBMN. Increased M/N, NBr/N and NBu/N indexes and a reduced B/N index were observed (Fig 3, lower panel), indicating serious damage to the ability of cells to successfully replicate. The mitotic index results (the proportion of cells in metaphase) revealed that MeHg provoked a significant increase (Fig 4, upper panel). Because the cellular viability and viable cells were similar to those of the control group (Fig 1), this result may indicate possible disturbance of the cell cycle. This effect was subsequently confirmed by the analysis of the profile of the cell cycle by flow cytometry. MeHg intoxication induced changes in the profile with a larger cell population in the S and G2/M phases and a smaller cell population in G1/G0 phases (Fig 4, lower panel). This altered profile of the cell cycle could be due to arrest in these phases provoked by a cyclostatic effect of the non-lethal concentration of MeHg. An effect like this must necessarily affect cell proliferation. Analyzing cellular viability after MeHg withdrawal, a significant decrease was detected 24 h and 48 h after the end of exposure, when compared to that of non-exposed cells (Fig 5, upper left panel). These data were confirmed by decreases in the number of viable cells (Fig 5, lower left panel) and the confluence of the culture (Fig 5, right panel) 24 h after the end of exposure.

## Discussion

This study demonstrated that exposure to a low, sublethal and non-apoptotic concentration of methylmercury causes significant genotoxicity and disturbances of the cell cycle and proliferation in cells of glial origin. Exposure of C6 cells to MeHg for 24 h showed an LC_50_ (the lethal concentration allowing 50% cellular viability) of 6.08 μM (Fig 2), a similar value to those previously described in other cell lines or primary cultures of CNS [8, 11, 21, 22]. Our results seem to be closer to those of primary cultures of human astrocytes exposed to this metal, compared to data using other glioma models in similar conditions [21, 23]. Although the MTT assay is widely used to evaluate cell survival, it is based on the detection of mitochondrial activity, which sometimes underestimates the initial processes of degeneration (such as early-stage apoptosis) [24]. However, results with the differential BE/AA staining method showed similar numbers of viable cells in both the control and exposed groups without an increased frequency of apoptotic or necrotic cells (Fig 2), confirming the MTT results.

Exposure to this low concentration of MeHg, allowing 100% of cellular viability without apoptosis, caused detectable genotoxicity, as revealed by an increase in all parameters of DNA damage (Fig 3). In cells of glial origin, significant DNA fragmentation (without detectable apoptosis) only was observed after treatment with 25 μM MeHg for 48 h [23], a higher concentration and longer incubation time than those used here (3 μM, 24 h). A possible explanation is that the astrocytoma cell line (CCF-STTG1) used by Pieper et al. may be more resistant to MeHg than primary cultures of human astrocytes [21]. CCF-STTG1 seems to be even more resistant than other astrocytoma cell lines, such as D384 [25]. Thus, our model may be closer to those of primary cultures, showing a similar LC_50_, as discussed.

Previously, we also demonstrated that exposure to MeHg concentrations as low as 1 μM provokes micronuclei and nucleoplasmic bridges in both human neuroblastoma and glioblastoma cell lines [11]. This work demonstrates that MeHg generates micronuclei, nuclear buds and nucleoplasmic bridges in glial cells, even in the presence of serum and with concentrations allowing 100% cellular viability (Fig 2). The frequency of micronucleated cells (MNC) in the control group (about 5%) was very similar to that previously described for other cell line of glial origin [11], thereby validating our model. This minimum MNC is present also in cells of the CNS, such as those of neuronal origin [11, 26]. However, this is the first time that a significant increase in MNC caused by such a low and sublethal MeHg concentration has been shown in glial cells (Fig 3).

Since the presence of nucleoplasmic bridges is related to events such as incomplete repair of the DNA strand or the presence of chromosomal rearrangements [27], an increased frequency of cells with nucleoplasmic bridges (NBr) could be associated with the DNA fragmentation, as observed in the Comet assay (Fig 3). Moreover, the NBr/MNC ratio [28] for our results was 1.75, suggesting the MeHg shows clastogenic characteristics at low sublethal concentrations in glial cells. These data confirm preliminary results with a human glioblastoma cell line where a clastogenic effect of MeHg was also noticed [11]. Supporting this hypothesis, a recent work has shown that MeHg intoxication inhibits the activity of the poly (ADP-ribose) polymerase-1 enzyme (PARP-1), which repairs breaks in the DNA strand [23]. MeHg intoxication also induced an increased proportion of cells with nuclear buds (NBu) in C6 glioma cell line (Fig 3). These structures are generated during gene amplification in the S-phase of the cell cycle, followed by the elimination of excess DNA [27]. Thus, in addition to DNA fragmentation, this low concentration of MeHg showed a deleterious effect on gene replication. This hypothesis may be also supported by a recent study reporting alterations in gene expression during exposure to this heavy metal [29]. Still, the values of NBr/MNC and NBu/MNC ratios (0.91 and 1.75, respectively) point to the formation of nucleoplasmic bridges as the main process responsible for the presence of micronuclei.

MeHg also reduced the proportion of binucleated cells (Fig 3), parameter that analyzes the number of cells that have correctly completed the cell cycle. This may be the first clue showing that MeHg, even under non-lethal conditions, is able to alter the cell cycle of glial cells. Reinforcing this idea, the mitotic index (MI) revealed an increase in cells in metaphase following mercury exposure (Fig 4). A similar increase (approximately 95%) was previously shown in a glioblastoma cell line [11]. Taken together, these data point to a change in the cell cycle that is consistent in different models of glial cells exposed to MeHg.

Analysis of the complete profile by flow cytometry revealed increased cell populations in the S and G2/M phases (Fig 4), confirming the disturbance of the cell cycle. Moreover, no significant change was observed in the fraction sub-G1 (this population might suggest the occurrence of apoptotic cells—[30]), confirming the data on viable cells. MeHg exposure has been associated with interferences in the cell cycle of different cells, especially neuronal cell lines and neuronal progenitor cells [12, 13, 15]. Although an increase of G1-phase cells due to MeHg exposure has been detected in some studies [13, 15], other studies have also reported an increase in cells in S or G2/M [12, 31]. These conflicting results with MeHg seem to be independent of the origin of the model (primary culture or cell line), since an increased population in the G2/M phase has been detected in cells of different origin than SNC, such as fibroblasts [32]. The diversity of the results seems to be more related to variations in the MeHg concentration, since changes in this parameter results in alterations to the cell cycle profile [33]. However, no data about the effect of MeHg on the cell cycle of glial cells is available; our work is the first to demonstrate that a sublethal concentration of this metal promotes alterations in the cell cycle profile with increased populations in the S and G2/M phases. An interesting fact is that despite the higher number of C6 cells in G2/M, only a small number of cells was able to reach cytokinesis (as revealed by the proportion of binucleated cells). Both effects of MeHg may indicate a reduction in cell proliferation. I*n vitro* studies, mainly using neural stem cells (NSC), showed that MeHg exposure causes decreased proliferation and impaired cell differentiation [34–36]. However, NSC show higher sensitivity to xenobiotic compounds than differentiated glial or neuronal cells [37]. In differentiated cells, MeHg intoxication has been demonstrated to be able to decrease the proliferation of cells of neuronal origin [13, 14]. Although previous studies have suggested that this event can also occur in glial cells [11, 23, 38], this effect was analyzed in this type of cells for the first time.

One important aspect of this experiment is that both dose (3 μM) and exposure time (24 h) to MeHg would be considered relatively safe when compared to the internationally established limits and, as may be expected, no changes in cellular survival may observed at the end of the exposure time. However, 24 h after the withdrawal of MeHg, cellular viability was significantly reduced (about 37%) when compared to the control group (Fig 5). In addition to these results, the significant decrease in the number of viable cells, the prolonged doubling time of the C6 cell culture and the diminished cellular confluence observed 24 h after the MeHg withdrawal confirm that these effects were caused by alterations in cell proliferation (Fig 5). Why has this difference in cell proliferation been observed only in this experiment and not before? Although additional studies are necessary, one possible explanation could be that, due to the low concentration of MeHg used here, a longer period of time is necessary to accumulate changes in DNA and the cell cycle (such as those triggered after 24 h of MeHg incubation with MeHg), leading to significant differences in the proliferation of cells of glial origin. One last important aspect is that although the cells were able to recover a similar profile of cellular proliferation (exponential growth observed 48 h after the end of MeHg exposure), the delay in proliferation registered 24 h after MeHg withdrawal (caused by the cytostatic effect of the metal during the exposure time) was maintained. These results suggest that the consequences on cellular proliferation are sustained beyond the effects registered at the end of the exposure.

Long-term effects of exposure to relatively low levels of mercury are still not well known, especially for chronic intoxication [4, 5, 8]. The main vehicle of chronic exposure to methylmercury is usually the fish consumption [4, 5]. A recent report demonstrated that mercury content in human brains was positively correlated with the number of seafood consumed per week [39]. Levels of 0.25–2.67 μg/g of mercury were showed in the midfrontal region, the inferior temporal region and the cerebellum of the participants of the latter cohort from Chicago [39]. The dose used in our work, 3 μM (equivalent to 0.75 μg/g, approximately), is between these values. Interestingly, it is below the levels found for occupational exposure in mercury mine workers: 10, 3 and 1.1 μg/g of mercury in the pituitary gland, nucleus dentatus and pineal gland, respectively [40]. These facts may suggest that the concentration used in our work is closer of brain concentrations detected with chronic exposure to relatively low levels of mercury, and below those found in occupational exposure. Moreover, concentrations of 2.5–10 μM of MeHg in the brain (estimated from human blood and hair mercury levels) have been associated with delayed psychomotor development in children and adults with minimal signs of MeHg poisoning [41–44]. Our results suggest that exposure to these low levels of mercury may lead to serious genotoxic consequences, additionally to the possible motor alterations. The importance of this finding contributes to the controversy established in the last decade regarding whether current limits for MeHg exposure are safe [9, 11, 45, 46]. Due to the multifactorial origin and long-term consequences of genotoxicity, it is difficult to definitively associate it to human exposure in epidemiological studies, especially with isolated populations such as in the Amazon region (with confounding factors such as endemic pathologies, miscegenation or the influence of occupational exposure, among others). However, a growing literature highlights genotoxicity as a major effect for MeHg, especially at low and relatively “safe” exposure levels.

Our work has demonstrated that exposure to a low sublethal concentration of MeHg (considered relatively safe, according to current limits) is able to promote genotoxicity and disturbances in the proliferation of cells of glial origin, with sustained consequences after MeHg withdrawal. This fact becomes especially important, since this cellular type accumulates more MeHg than neurons and displays a vital role protecting the CNS, especially in chronic intoxication with this heavy metal.
